# A TmaT-AftD interaction is required for the CmpL4 mycomembrane biogenesis pathway in *Corynebacterium glutamicum*

**DOI:** 10.64898/2026.07.31.741767

**Published:** 2026-07-31

**Authors:** Anastacia R. Parks, Eric D. Snow, Victoria M. Marando, Michael J. James, Vincent de Bakker, Laura L. Kiessling, Suzanne Walker, Thomas G. Bernhardt

## Abstract

Bacteria in the Mycobacteriales order like mycobacteria and corynebacteria surround themselves with a multilayered cell envelope. Their cytoplasmic membrane is fortified by a peptidoglycan cell wall that is decorated with branched arabinogalactan (AG) polymers. The AG glycans are further modified with mycolic acids to form an outer membrane. Biogenesis of this mycomembrane requires the transport of mycolates from their site of synthesis in the cytoplasmic membrane to the cell surface. How mycolate transport is controlled and coordinated with AG synthesis has remained unclear. Mycolate transport is mediated by the essential RND-family transporter MmpL3 in mycobacteria and a pair of related, partially redundant transporters called CmpL1 and CmpL4 in corynebacteria. The acetyltransferase TmaT has also been implicated in mycolate transport in both types of bacteria. In corynebacteria, it is required for the production of acetylated mycolates, and this modification has been proposed to promote mycolate transport via both CmpL transporters in the model organism *Corynebacterium glutamicum* ( *Cglu* ). Here, we reinvestigated the function of TmaT in *Cglu* and found that it and several factors encoded in the *tmaT* locus are specifically required for mycolate transport via the CmpL4 transporter pathway. Notably, one of these additional genes encodes the arabinosyltransferase AftD involved in AG biogenesis. TmaT and AftD were found to interact, and our results indicate that this interaction is required for acetylated mycolate production, mycolate transport via the CmpL4 pathway, and normal arabinan synthesis. Thus, the TmaT-AftD interaction may serve as a regulatory link connecting mycolate transport with AG biogenesis.

**SIGNIFICANCE:**

Mycobacteriales bacteria, including pathogens like *Mycobacterium tuberculosis* ( *Mtb* ), have a complex cell surface comprising an inner membrane, a cell wall modified with arabinogalactan (AG), and an outer mycomembrane made of mycolic acids linked to AG polymers. Because these surface biogenesis pathways are targeted by frontline anti- *Mtb* drugs, there is great interest in elucidating their underlying mechanisms. Here, we identify an interaction between factors involved in mycomembrane (TmaT) and AG biosynthesis (AftD) in the model organism *Corynebacterium glutamicum.* We show that this interaction is important for proper surface biogenesis and may therefore function to coordinate mycomembrane assembly with AG synthesis. This and other potential regulatory connections controlling envelope biogenesis represent attractive targets for future antibiotic development.

## Full Text Availability

The license terms selected by the author(s) for this preprint version do not permit archiving in PMC. The full text is available from the preprint server.

